# Fundamental differences in patterns of retinal ageing between primates and mice

**DOI:** 10.1038/s41598-019-49121-0

**Published:** 2019-08-29

**Authors:** Jaimie Hoh Kam, Tobias W. Weinrich, Harpreet Shinhmar, Michael B. Powner, Nicholas W. Roberts, Asmaa Aboelnour, Glen Jeffery

**Affiliations:** 1 University College London, Institute of Ophthalmology, EC1V9EL London, UK; 2 City, University of London, Centre of Applied Vision Research, EC1V0HB London, UK; 3 School of Biological Sciences, University of Bristol, BS8 1TQ Bristol, UK; 4 Histology and Cytology Department, Faculty of Veterinary Medicine, Damanhour University, Damanhour, Egypt

**Keywords:** Retina, Experimental models of disease

## Abstract

Photoreceptors have high metabolic demands and age rapidly, undermining visual function. We base our understanding mainly on ageing mice where elevated inflammation, extracellular deposition, including that of amyloid beta, and rod and cone photoreceptor loss occur, but cones are not lost in ageing primate although their function declines, revealing that primate and mouse age differently. We examine ageing primate retinae and show elevated stress but low inflammation. However, aged primates have a >70% reduction in adenosine triphosphate (ATP) and a decrease in cytochrome c oxidase. There is a shift in cone mitochondrial positioning and glycolytic activity increases. Bruch’s membrane thickens but unlike in mice, amyloid beta is absent. Hence, reduced ATP may explain cone functional decline in ageing but their retained presence offers the possibility of functional restoration if they can be fuelled appropriately to restore cellular function. This is important because as humans we largely depend on cone function to see and are rarely fully dark adapted. Presence of limited aged inflammation and amyloid beta deposition question some of the therapeutic approaches taken to resolve problems of retinal ageing in humans and the possible lack of success in clinical trials in macular degeneration that have targeted inflammatory agents.

## Introduction

The central nervous system (CNS) has high metabolic demand and this contributes to the pace of ageing as energy consumption and ageing are linked at the level of the organism and the organ^1–5^. Here the retina stands out as it has the greatest energy demand in the body due to the high metabolic rate of its photoreceptors^6^. The consequence is that cellular ageing in the outer retina is marked^7^, and associated in humans with a significant decline in visual function decades before average life expectancy is reached^8–10^.

Key features of retinal ageing have been established in studies using rodents. These describe progressive inflammation^11^ and deposition of extracellular material including amyloid beta in the outer retina^12^. This is associated with photoreceptor loss established in the first year in the cone population and later in rods^13–15^. Mitochondria may play a key role in such events as they produce adenosine triphosphate (ATP) that fuels metabolic activity. Mitochondria are densely packed in photoreceptor inner segments and their ATP production declines with age, undermining function^16,17^. They also have the ability to signal cell death^18^, which becomes a key feature of the aged outer retina with a 30% photoreceptor loss in the rod population^7,13,14^.

In spite of many studies, significant questions arise regarding the relevance of rodent models to human retinal ageing. Rodents are nocturnal and avoid light preferring to live in darkness. The majority of primates, however, are diurnal and their visual systems are fundamental to their ecology and behaviour. They have evolved with a larger cone population concentrated in a macular region where retinal cell density peaks^19^. Furthermore, primate and rodent retinae age differently, with no evidence for cone loss in primates^7,20^. This is mirrored in humans, where cones on which human vision is almost entirely dependent, are highly resistant to cell death. Even in age related macular degeneration (AMD) where there is progressive central atrophy, cones persist at the margin of the atrophic region when surrounding rods have died^7^. Their survival may be linked with the specific accumulation of phosphorylated tau in their inner segments that likely undermines mitochondrial function^21^, but which also blocks cell death by reducing cytochrome c release and caspase activity^22^. As cell death is an end point in ageing, such fundamental differences likely reflect mechanisms of ageing that are very different between primates and mice.

We investigate retinal ageing in an old world primate *Macaca fascicularis* that has a retina highly similar to the human with a well-developed macular and fovea. We reveal key features and mark how they differ significantly from mouse models, questioning the relevance of these models for human retinal ageing research.

## Results

### Protein arrays for stress and inflammation

Changes in stress related and cytokine proteins were measured between young and old primates in the macular and the periphery. Stress markers were measured in retina and cytokines in retina and in the RPE/choroid complex. Stress markers were not measured in RPE/choroid because they are poorly expressed here.

### Stress marker arrays

**Figure 1:**
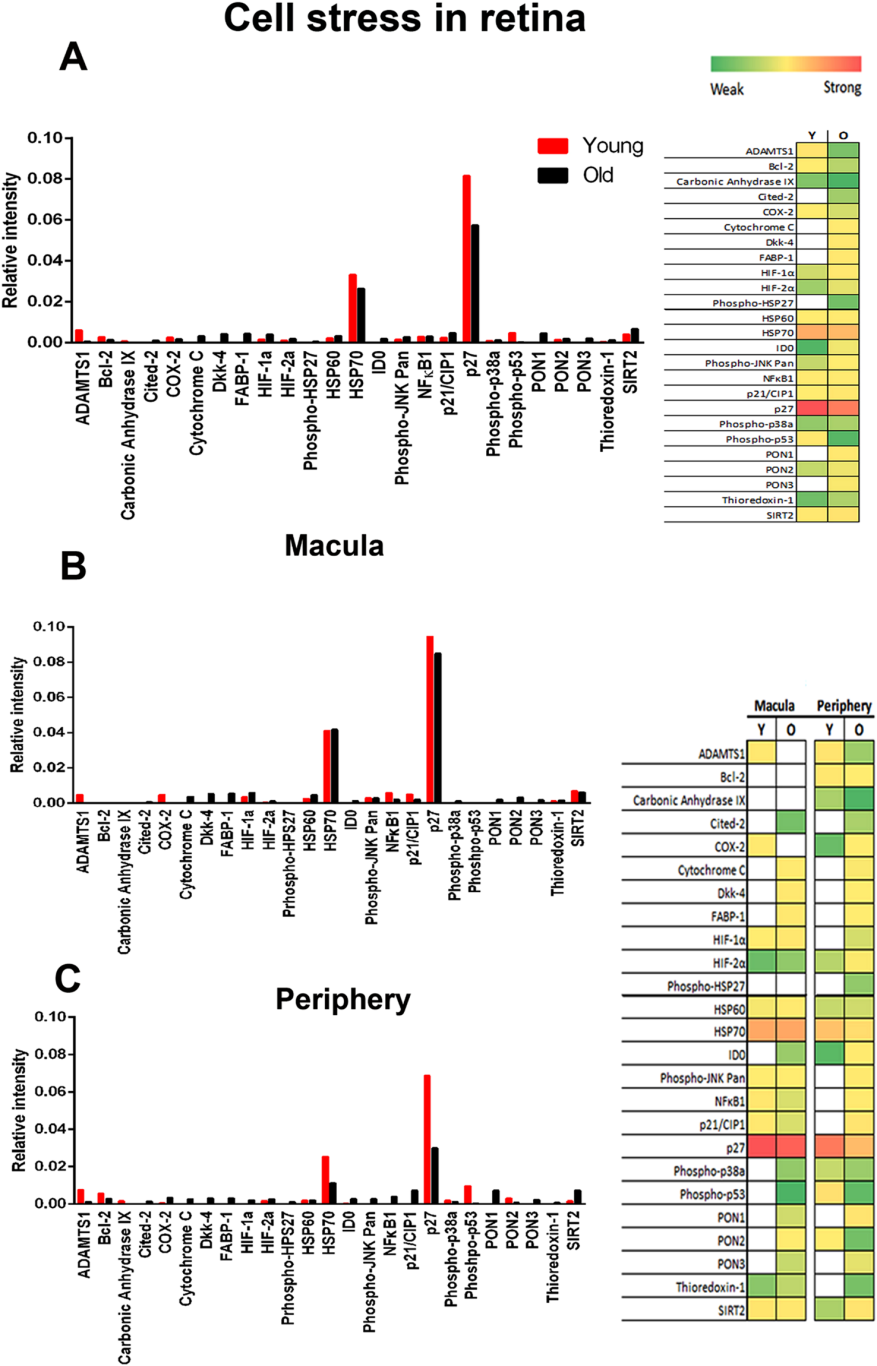
Stress markers in primate retina. (**A**) Primate proteome cell stress array from young and old primates for total retina with heat map representation to the right. Data show a small overall upregulation of a number of stress markers with age. Of the 25 stress proteins 17 were up regulated in older animals, which are seen in both the main graph and heat map representation. The main plot is dominated by two principal components, HSP70 and p27, neither of which showed an age related increase. Six proteins were clearly expressed at higher levels in the younger than the older animals. (**B**,**C**) Arrays were divided into those from macular and periphery. Patterns in both are similar to total samples seen in A. Hence age related stress increases relatively uniformly across the retina. On the right is a heat map for these data reflecting relative overall changes. White represents not detected. Green is weak expression and red is strong expression in Young (Y) and old (O). Supplementary Data provides raw scores for the heat maps. N = 5 in each group, with tissue being pooled. Average age of young is 4 years and 2 months, SD ± 2 months and for old primates is 14 years and 7 months, SD ± 4 months.

These showed clear age related increases between young and old primates for the 26 proteins examined (Fig. 1). In the macular and periphery, more stress markers are upregulated in old compared to young animals (Fig. 1). In the macular 10 stress markers are upregulated in young primates and 17 in old animals. In the peripheral regions 11 stress markers are found in young retinae and 22 in old. Hence, ageing is associated with increased retinal stress. The principal components of the stress arrays, P27, and HSP70 were greater in the macular than the periphery for both young and old primates. Although most of the 26 cell stress related proteins were upregulated in the older primates, some were also downregulated most notably, ADAMTS1, Bcl-2, Carbonic Anhydrase IX, COX-2, HSP70, p27 and phospho-p53.

### Cytokine markers

**Figure 2:**
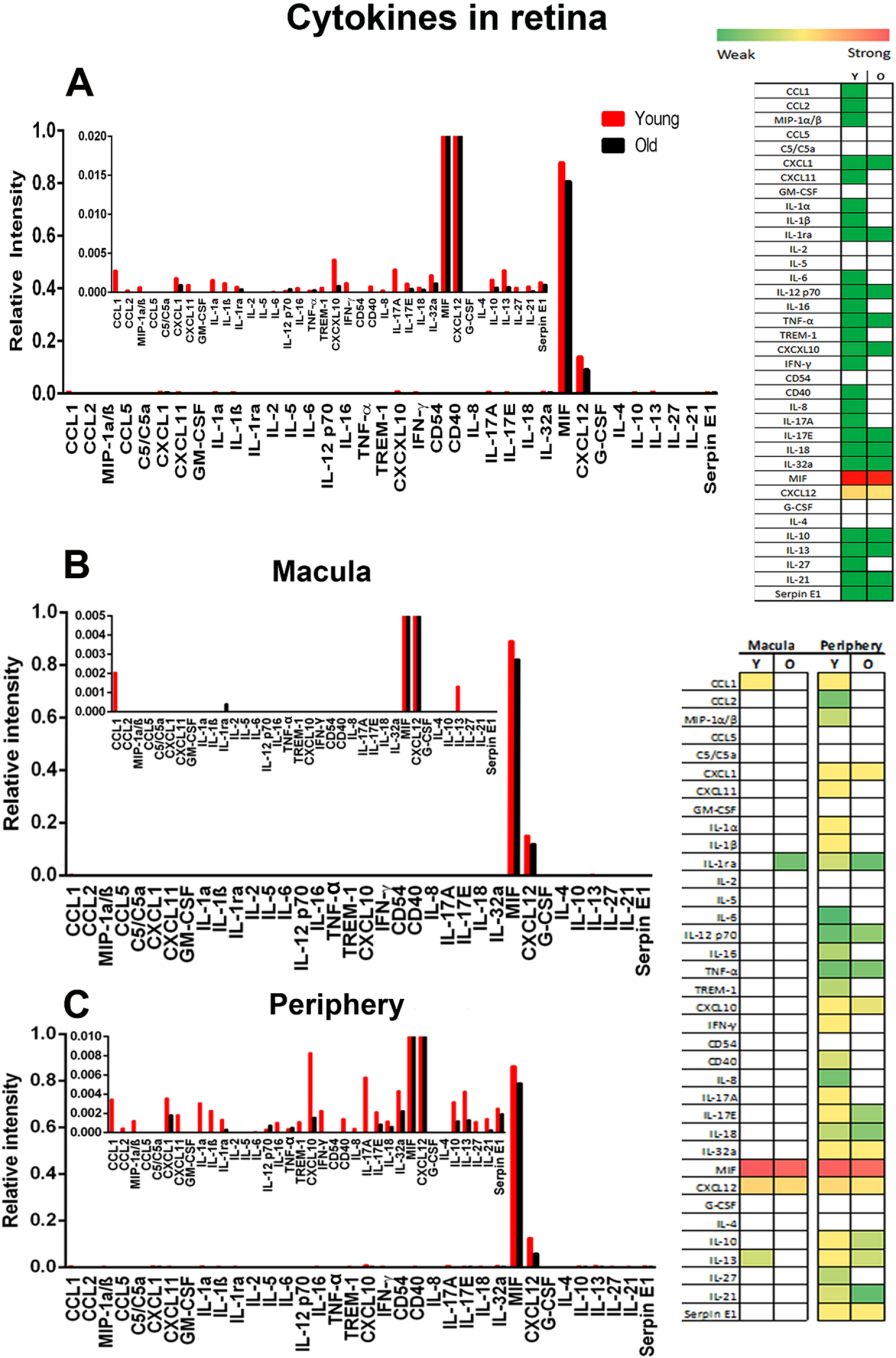
Cytokine expression in primate retina. (**A**) Cytokine arrays from young and old primates for total retina with heat map representation to the right. There was very low cytokine expression and no marked general increase with age. Only MIF and CXCL12 were expressed at significant levels, but even here there was no age related increase. In the insert the Y axis has been re-scaled to reveal cytokines expressed at a lower level with MIF and CXCL12 truncated. (**B**,**C**) Arrays were divided into macular and periphery. Patterns in both are similar to total levels shown in A. However, when the data are rescaled for each region in the inserted graphs higher levels of cytokine expression over a broader number of cytokines are seen in the periphery. On the right is a heat map for these data reflecting relative overall changes. White represents not detected. Green is weak expression and red is strong expression in Young (Y) and old (O). Supplementary Data provides raw scores for the heat maps. N = 5 in each group with tissue being pooled. Average age of young is 4 years and 2 months, SD ± 2 months and for old primates is 14 years and 7 months, SD ± 4 months.

Ageing in mice is normally characterised by a proinflammatory state that can initiate age-related diseases^11,18^. However, our results reveal little if any up regulation of these cytokines in primate retinal arrays (Fig. 2). Only two cytokines were clearly expressed, MIF and CXCL12.

A wider range of cytokines are expressed in the peripheral retina than the macular (Fig. 2), but these remain at relatively low levels and are only revealed when the scale of the Y axis is expanded. However, even here there is no overall increase in expression with age. Data for the macular and the peripheral imply that these regions are different and that the macular possesses a degree of protection from cytokine based inflammation that is not seen in the periphery.

**Figure 3:**
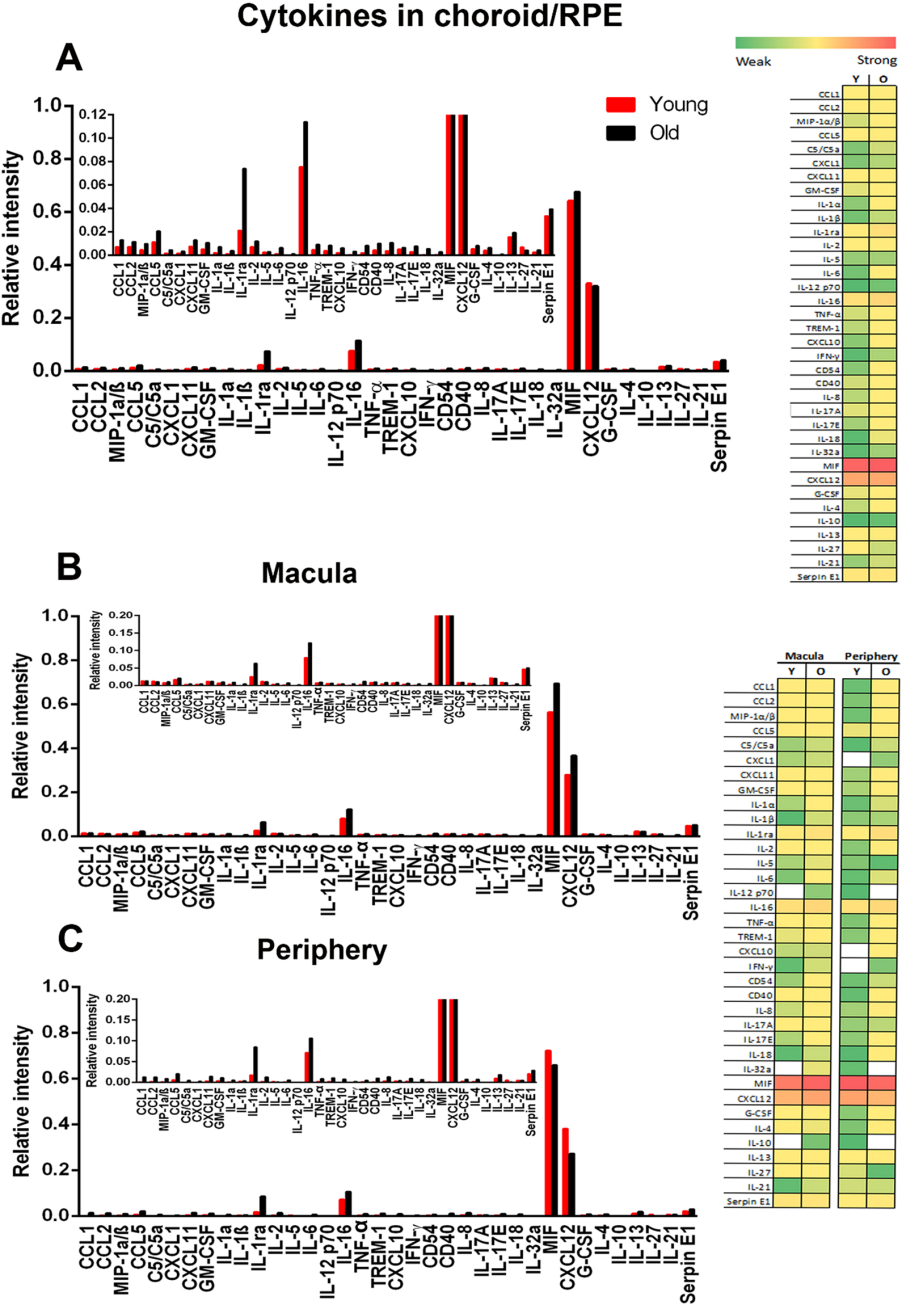
Cytokine expression in primate choroid/RPE. (**A**) Cytokine expression in primate RPE/choroid complex in young and old animals. While cytokine expression was low in both age groups there was an overall increase with age revealed in both graphs and heat maps. As with the retina the graphs are dominated by MIF and CXCL12 expression. The insert is re-scaled to show low level expression with MIF and CXCL12 truncated. (**B**,**C**) show data derived from macular and peripheral regions, which generally mirror data for total retina. Again in each data are rescaled in inserts with MIF and CXCL12 truncated. On the right is a heat map for these data reflecting relative overall changes. White represents not detected. Green is weak expression and red is strong expression in Young (Y) and old (O). Supplementary Data provides raw scores for the heat maps. N = 5 in each group with tissue being pooled. Average age of young is 4 years and 2 months, SD ± 2 months and for old primates is 14 years and 7 months, SD ± 4 months.

A cytokine array analysis was undertaken on the primate RPE/choroidal tissue (Fig. 3). This reveals marginally greater levels of expressions than found in the retina and a shift towards an age related increase. MIF and CXCL12 were again highly expressed in the RPE/choroid tissue, reflecting the data obtained from the retina (Fig. 2).

**Figure 4:**
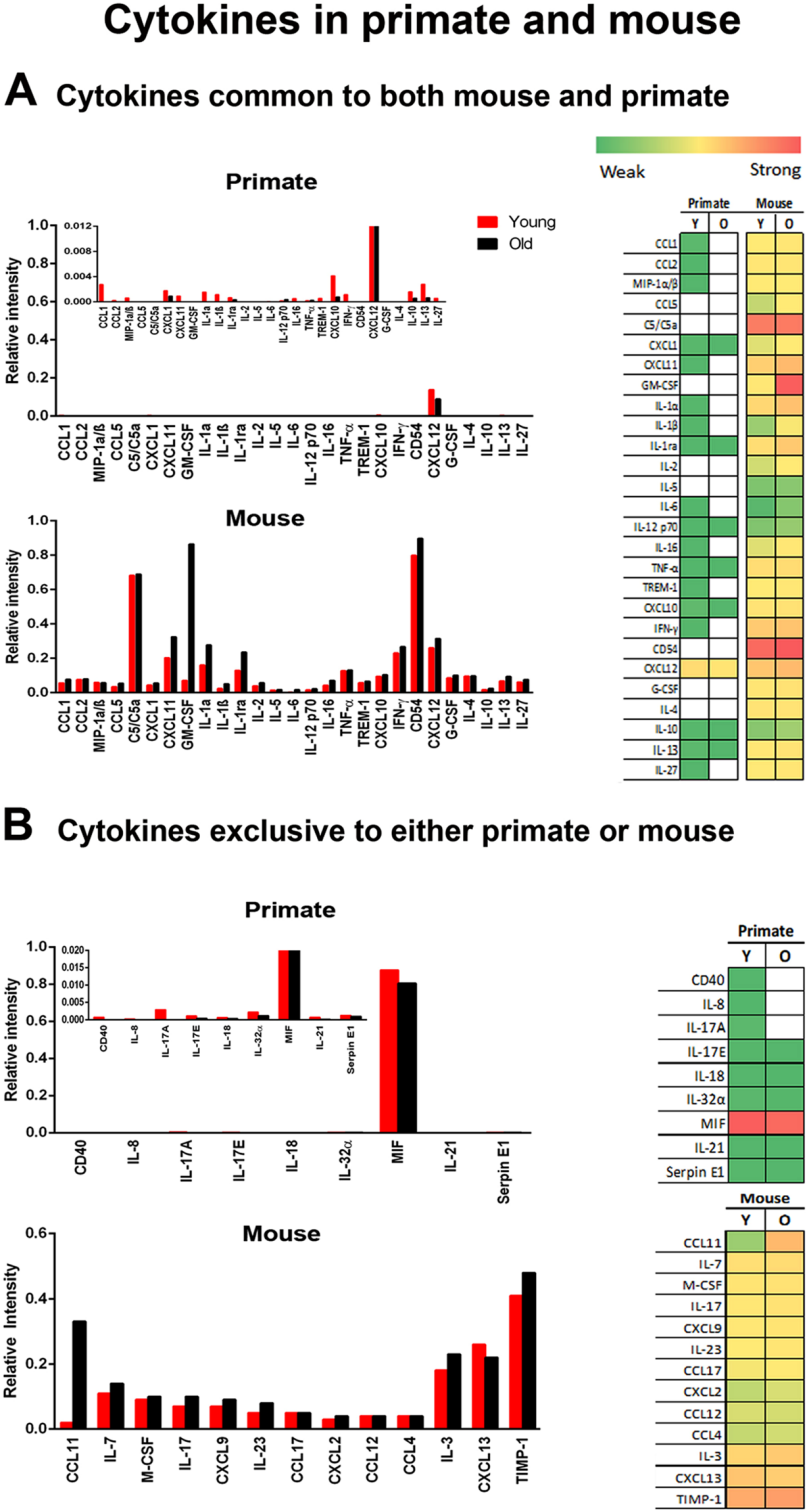
Expression of retinal cytokines in primate and mouse. (**A**) Those common in both primate and mouse. Here cytokine expression is much higher in mice than primate and in mouse shows an age related increase in many. Because expression is so low, the primate data have been rescaled by 2 log units in the insert with CXCL12 truncated for comparison. (**B**) Cytokines that were exclusive to either primate or mouse. Again, much higher levels of expression are found in mouse than primate and in mouse there is a generally an age related increase in expression. Primate patterns are dominated by MIF and data has been rescaled in the insert with MIF truncated for comparison. There is no age related increase in expression in primate. On the right is a heat map for these data reflecting relative overall changes. White represents not detected. Green is weak expression and red is strong expression in Young (Y) and old (O). Supplementary Data provides raw scores for the heat maps. N = 5 in each group with tissue being pooled. Average age of young is 4 years and 2 months, SD ± 2 months and for old primates is 14 years and 7 months, SD ± 4 months. N = 6 per group of mice. Young mice were 2 months and 12 months for old.

Much of current understanding of aged and diseased retinal inflammation derives from research on mice^11^. To determine species differences between primates and mice we directly compared relative cytokine levels in ageing for those cytokines commonly expressed in the retinae of both species (Fig. 4A). Differences between species are marked, with much greater levels of cytokine expression in mice than primates. Further, in mice many of these are clearly up regulated with age. While CXCL12 is a principal component in primates, its relative level of expression is much lower in mice. In mice the principal components are CD54, GM-CSF and C5/C5a, which all show aged increases. While these are present in primate, they are expressed at much lower levels and show no aged increase.

Array analysis was then undertaken for cytokines that were only expressed in either primates or mice (Fig. 4B). These reflect the overall pattern seen in Fig. 4A, with many more cytokines expressed in mice than primates. In primates the principal component was MIF that did not show an age related increase. When other cytokines expressed at much lower levels were revealed in the insert in Fig. 4 where the Y axis is expanded, none showed an age related increase. However, of the 13 cytokines identified in mice, 9 showed age related increases. Hence, overall mice have much greater levels of cytokine expression in the retina and show clear age related increases not seen in primates.

### ATP production

**Figure 5:**
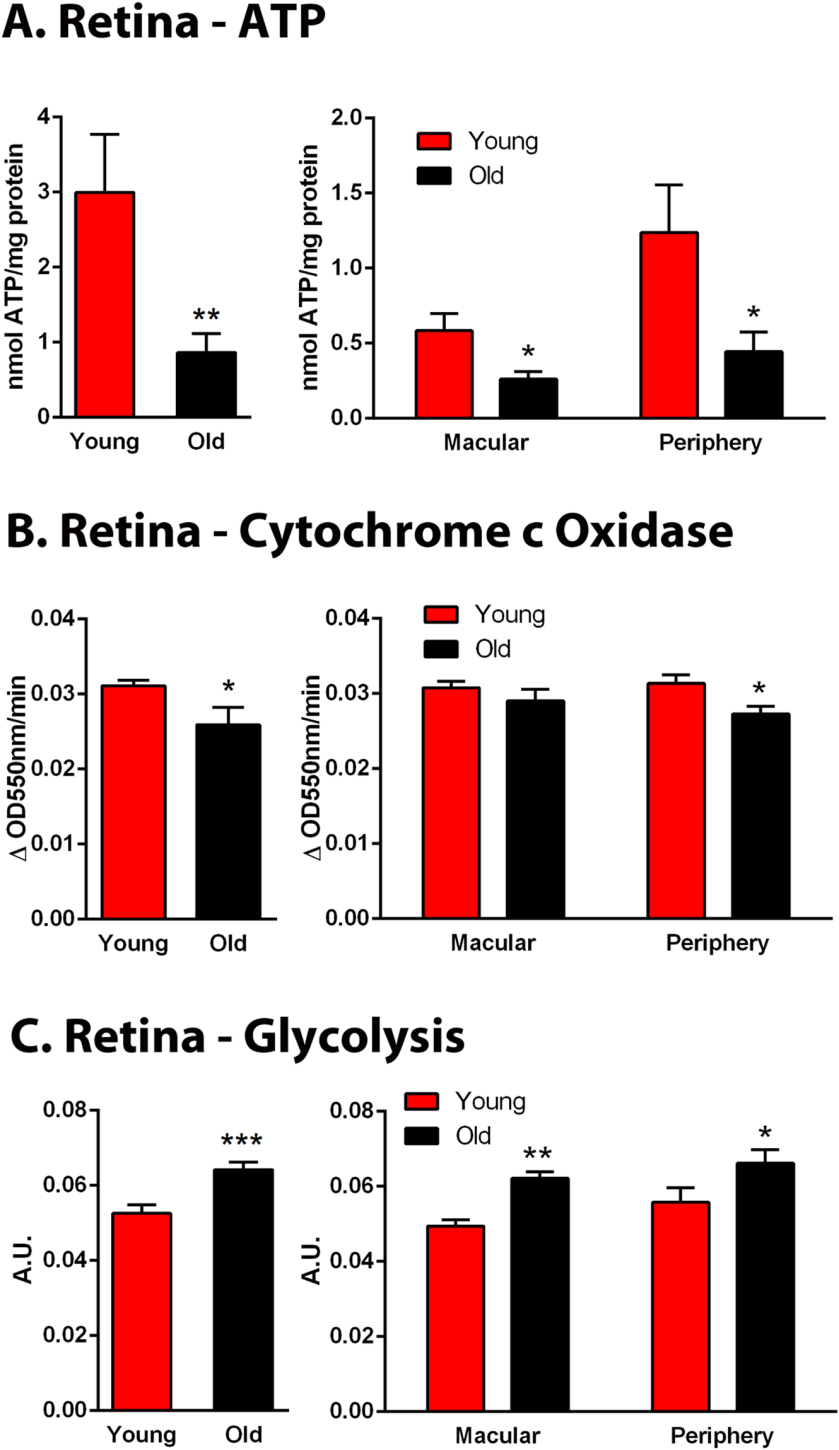
Age related changes in primate retinal metabolism. (**A**) Retinal ATP was measured in whole retinae and from both macular and periphery in young and old primates with data normalised for protein content. With age there was a significant >70% fall in the overall ATP concentration of the retina. When age related changes were divided into those from the macular regions and those from the periphery significant reductions were found in both. (**B**) At the same time there was a significant increase in glycolysis of approximately 20%. Again this was present in both the macular and the periphery. (**C**) Cytochrome c oxidase activity as measure at the same ages. Overall there was an approximate 20% decline with age across the retina although when samples were taken from the macular and the periphery the decline was only statistically significant in peripheral regions. N = 5 in each group. Average age of young is 4 years and 2 months, SD ± 2 months and for old primates is 14 years and 7 months, SD ± 4 months. ***P ≤ 0.001, **P ≤ 0.01 and *P ≤ 0.05.

The array data reveal that stress is present in the aged primate but is not associated with elevated inflammation. However, such stress could result from reduced mitochondrial activity and declining ATP production, undermining normal physiological function. Hence, ATP levels and cytochrome c oxidase activity were measured (Fig. 5A,B). ATP was reduced by >70% in aged primates. Analysis was then divided into measurements taken from macular and periphery. In the macular ATP was reduced by 60% while in the periphery reductions were approximately 80%. These changes were reflected in a 15% reduction in the activity of cytochrome c oxidase (Fig. 5B). Reductions in cytochrome c oxidase were present in both the macular and the periphery, although only in the periphery was it significant.

In ageing it has been assumed that mitochondrial decline may be associated with a compensatory increase in glycolysis^23^. However, this carries a high price because glycolysis is associated with an increase in the pace of ageing^24^. ATP can be generated by mitochondrial respiration or glycolysis, although the glycolytic pathway is relatively inefficient producing only 2 molecules of ATP per molecule of glucose compared with 36 molecules of ATP for mitochondrial respiration. Glycolysis increased significantly by approximately 20% overall in aged primate retinae compared to young animals (Fig. 5C) and this significant increase was present in both macular and periphery equally. Hence, overall ATP levels fall markedly with age even though glycolysis is upregulated, implying that aged mitochondrial dysfunction may be greater than that reflected by ATP production alone.

### Mitochondrial morphology

**Figure 6:**
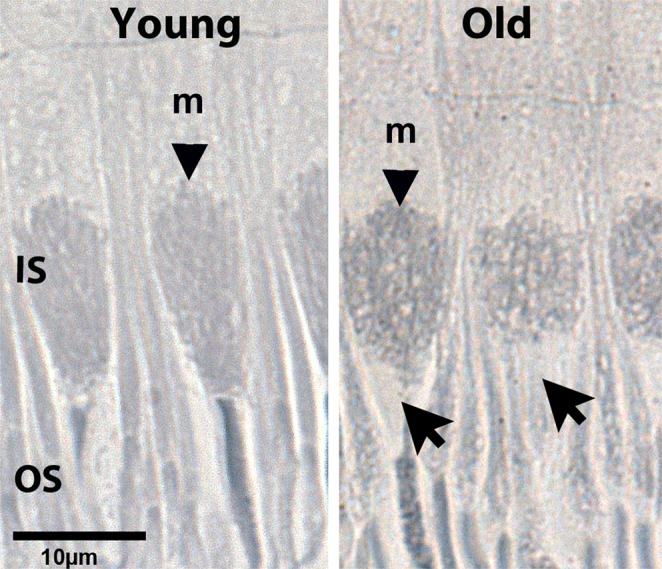
Age related changes in primate mitochondrial alignment. Retinae from young and old primates were osmicated, embedded in plastic, sectioned and stained with Sudan Black B. This marked lipid content including that found in mitochondria in inner segments (IS) and that present in outer segments (OS). Outline images of mitochondria (m) in IS in the two age groups were different in cones. In young animals they reflected patterns commonly found with mitochondria roughly aligning along the long axis of the IS. In older animals this pattern was lost and many mitochondria appeared to be aligned orthogonal to this axis as can be seen by the appearance of profiles with a circular appearance. Also in older primates marked gaps were present at the junction of the IS and the OS that were now mitochondria free (Arrow). N = 5 in each group. Average age of young is 4 years and 2 months, SD ± 2 months and for old primates is 14 years and 7 months, SD ± 4 months.

Because there are such large reductions in ATP in aged primate retinae, we ask if this is reflected in differences in mitochondrial organisation in photoreceptor inner segments. By combining two lipid staining techniques, using osmium tetroxide and Sudan Black B, mitochondrial outlines can be identified at the light microscope (LM) level in thin plastic sections. This method affords the ability to examine a relatively large number of cells compared with an analysis at the electron microscope level. Mitochondrial staining patterns were very different in young and old primates (Fig. 6). In young primates images were consistent with classic descriptions, with mitochondria in ellipsoid regions of inner segments of both rods and cones aligned along the cells long axis^25^. This was not the case in the old animals where many mitochondria were seen in cross section producing circular outlines. In cones these were within relatively enlarged inner segments. This pattern is also present in the rods. Further, within aged cones, mitochondria commonly failed to populate the distal regions of the inner segment adjacent to the outer segment (arrows Fig. 6).

### Thickness of Bruch’s membrane and extracellular deposition

**Figure 7:**
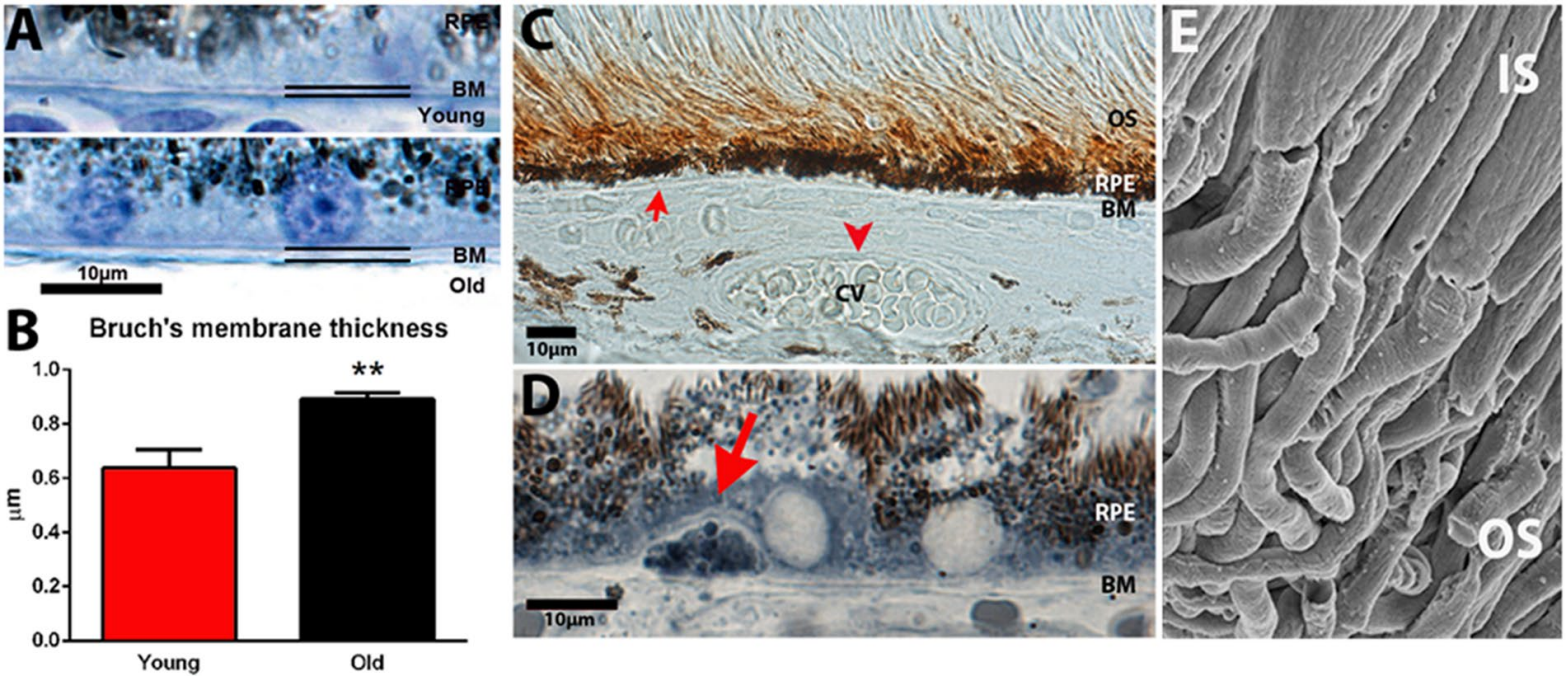
Age related changes in primate outer retina. There were age related changes in association with Bruch’s membrane (BM). (**A**,**B**) BM became significantly thicker with age by approximately 40%. (**C**) Amyloid beta is a component of BM deposition and present in association with outer segments in mice and has been reported on human outer segments. Staining for Aβ (brown) revealed its presence in young and old primates alike on outer segments, but in no animal was it present on BM (red arrow) or in association with choroidal vessels (cv red arrow head). This absence is in contrast to age mice. (**D**) Only rarely were there any deposits on BM with an appearance similar to human drusen, but these were small remaining under the RPE. They were Aβ negative but they did stain with Sudan black B (red arrow). (**E**) Scanning electron microscope image from age primate outer retina. There were no obvious deposits on outer segments (OS). Inner segments (IS) are to the top. N = 5 in each group. N = 3 for E. Average age of young is 4 years and 2 months, SD ± 2 months and for old primates is 14 years and 7 months, SD ± 4 months. **P ≤ 0.01.

If ATP production is reduced this may be correlated with increased thickness of Bruch’s membrane resulting in reduced outer retinal perfusion, which would restrict the oxygen supply critical in ATP production. Measurements of membrane thickness showed a 40% increase with age (Fig. 7A,B).

A key element in Bruch’s membrane deposition in aged mouse retina is amyloid beta that is deposited linearly on the membrane, in choroidal blood vessels and is also a component of deposition on photoreceptor outer segments. In human it is known to be present on outer segments^12,26^. However, there is little evidence for its general accumulation on the membrane in aged human retinae, although it is a component of drusen that are focal deposits^27^.

We stained primate sections with amyloid beta 4G8 similar to Hoh Kam *et al*.^12^ confirming that positive staining was present on outer segments (Fig. 7C). This antibody labels amyloid precursor protein and also the heavy oligomers amyloid beta (1–42). However, in spite of the 40% increase in Bruch’s membrane thickness we found no evidence for amyloid beta (4G8) as component of the membrane. Staining was also undertaken with two additional amyloid antibodies, DE2B4 and EPR1878 to examine components on Bruch’s membrane, and again both were negative (data not shown). Hence, we find no evidence for amyloid beta deposition on Bruch’s membrane in primates. Likewise we found no evidence for deposition of this material on the walls of choroidal vessels (Fig. 7C). We also found almost no evidence for focal deposition of any nature on Bruch’s membrane. Structures similar to drusen were very rarely seen (Fig. 7D), but when identified they were relatively small and did not rupture through the RPE as found in the aged human retina^27^. These small deposits stained heavily with Sudan black B implying that they had high lipid content.

In aged mice, scanning EM images of outer segments reveal material deposited upon them that is associated with amyloid beta staining^12^. When similar images similar to those in mice were examined from primates with scanning EM there was no evidence of extracellular deposition at any age (Fig. 7D). Taken together, these data imply that staining for amyloid beta with 4G8 identified a potentially functional protein in the membrane of outer segments rather than an extracellular deposit.

### Outer segment refractive error

In spite of the failure to reveal changes in outer segment deposition there were changes in the refractive error of outer segments consistent with age related changes in their structure. The phase retardation per micron of the photoreceptors was found to be significantly greater in outer segments of the younger animals compared to the older group (two sample t-test; t = 2.74, df = 18, p = 0.014). This increase in the retardation values was due to a greater refractive index of the outer segment. The greater refractive index of the outer segments found in the younger photoreceptors has the optical effects of the both increasing the effective numerical aperture of the outer segment and increasing the fraction of the optical power that can be guided into the cell.

## Discussion

We show that while ageing primate retinae suffer increased cellular stress, they do not suffer from cytokine driven inflammation and extracellular deposition, distinguishing them from ageing processes in mice^11,12^. In primates the key feature of aged decline is the significant reduction in mitochondrial function and ATP production that reduces available cellular energy in a metabolically demanding environment. Even though glycolysis increased significantly with age, it did not compensate for this decline. Overall ATP production was reduced by >70%, which is greater than found in old mice where reduction is only around 30%^16^. Given the high energy demands of the outer retina^6^, reductions of this magnitude are likely to impact on retinal function. As primates age, their photoreceptor mitochondria also show different distribution patterns appearing to lose their alignment along inner segment long axis adjacent to their oxygen supply^25^. Mitochondria are key regulators of ageing and also signal cell death when their membrane potential declines, and this may underpin the significant reduction in rod numbers with age^18^. However, there is no evidence for reduced cone numbers with age in primate, although these cells show functional decline^7,20^. Their survival may be due to the accumulation of phosphorylated tau, which although associated with mitochondrial decline has also been shown to be neuroprotective^21,28^.

Our results do show a relatively small aged increase in inflammation in primate RPE/choroid complex, which may be significant because this is where age related macular degeneration (AMD) is initiated^29^. It is also a region where in AMD there are fundamental changes in metabolism with reduced mitochondrial function^30^.

Some of the cell stress markers that were up regulated in primate are known to have specific functions or associations. HSP70 proteins are central components of the cellular network of molecular chaperones and catalysts helping in protein folding and also interacting with key regulators of signal transduction pathways controlling homeostasis, proliferation, differentiation and cell death. The functions of HSP70 are based on its ability to interact with proteins in an ATP-controlled fashion^31,32^ and this may explain why HSP70 is downregulated in the old primates as with age retinal ATP declines (See Fig. 5).

Phosphorylated p53 plays a role in cell cycle control and apoptosis. Feng *et al*.^33^ have found that p53 function declines with age, which reflects our results. In older retinae, the level of phosphorylated p53 is downregulated in the periphery but was not detected in the macula.

While it is not always obvious what role each stress marker may play, key markers are apparent with roles appropriate for their age. ADAMTS1 is associated with growth and anti-angiogenesis and is higher in young animals. Free cytochrome C is associated with cell death and is higher in older animals where significant rod photoreceptor loss is likely^7^. DKK-4 is associated with neurodegeneration and present at higher levels in older animals, particularly in the macular. Hypoxia-inducible factor (HIF) that is up regulated in the ageing macular. Here, increased hypoxia is a likely consequence of age related to Bruch’s membrane thickening that compromises outer retina perfusion (See Fig. 7). Bcl-2 is neuroprotective and more highly expressed in young.

Only a very limited range of cytokines were upregulated in aged primate retinae. MIF (Macrophage migration inhibitory factor) is an innate immunity molecule acting as both a pro- and anti-inflammatory agent regulating proliferation and survival. It is ubiquitous expressed particularly in the CNS^34,35^. CXCL12 is a homeostatic chemokine^36^, regulating T and B lymphocyte survival and generation of T cell memory^37^. CXCL12 also regulates homing/mobilisation of hematopoietic stem cells between bone marrow and peripheral blood^38^. It modulates neurotransmission, neurotoxicity and neuroglial interactions^39^. CXCL12 and its receptor CXCR4 are expressed in developing and mature CNS, but do not mediate leukocyte recruitment^39,40^, which may partially explain increased CXCL12 levels in the young compared to old retina.

The failure to find significant increases in aged cytokine driven retinal inflammation in primates stands in contrast to the mouse data. This may be a consequence of multiple factors. Here, the immune status of laboratory mice may play a key role. Mice have different patterns of immunity from primates and these can be exacerbated by being kept for many generations in specific pathogen free environments and intensely inbred. The C57Bl/6 mouse has been inbred since approximately 1921^41–46^. It has a very high incidents of microphthalmia, with up to 9% of animals displaying this feature, mainly in the right eye of females^47^. However, the primates used here had a very low inbreeding rate, probably similar to that in the wild, and were housed in an open environment. When such issues are combined with the very different patterns of retinal architecture between mice and primates, with the primate having major regional differences in cell density not seen in mice^19^, the validity of the mouse model for retinal ageing in humans becomes questionable. The legitimacy of mouse models for human disease is now widely questioned, particularly in relation to patterns of immunity^41,44,45,48^ and our results support this.

In comparing cytokine expression in mouse and primate, it is not only that there are such marked differences in the levels of cytokines, but also that in primates expression was heavily focussed on only two proteins, MIF and CXCL12. The reason for this is unclear, but this perhaps marks them out as of particular interest.

The absence of amyloid beta on Bruch’s membrane is another key difference separating primates and mice. Labelling for amyloid beta 4G8 that includes the precursor protein is present in association with primate outer segments, but not on Bruch’s membrane or in the lumen of choroidal vessels as found in mice^12,26^. Because of our failure to identify label with 4G8 we also used 2 other separate antibodies, but consistent with 4G8, these were also negative. Differences between mice and primates may imply that primate RPE is better able to degrade this material than in mice, and that this avoids deposition on Bruch’s membrane. Further, we were unable to identify deposited material on primate outer photoreceptor segments at the EM, which are present in mice^12^. However, there are age related changes to outer segments as revealed by changes in the reflective error of individual outer segments. This implies that something is changing in their structure with age, although the nature of this change is unclear.

Data presented here are consistent with the notion that amyloid beta detected in primate is likely to be a functional protein rather than a cytotoxic deposited material. Lighter amyloid beta oligomers are functional and modulate synaptic plasticity. Their removal has been linked to cognitive decline^49,50^. Synaptic plasticity is a feature of photoreceptor ribbon synapses^51–54^ and amyloid beta removal has been associated with reduced retinal responses^55^. Amyloid beta is found as a component of drusen in human retinae, but this is a relatively minor element of their volume^56^. Further, it is not deposited linearly along Bruch’s membrane as in mice where it is coincident with aged inflammation^12,57^, which is a significant species differences.

An important difference between the data presented here and that found in humans is that we rarely encountered drusen type deposits. When seen they were small and did not rupture through the RPE. However, our primates were maintained on healthy diets that were relatively low in fat and avoided the multiple variables that confound many human studies on retinal ageing, including diet, smoking and exercise. This difference may be important when looking for drivers of aged retinal disease. A high fat diet is a known risk factor for age related macular degeneration^58,59^. It is possible that primates maintained on different diets may have different retinal signatures in terms of inflammation and deposition.

With age a mitochondrial free region develops at the outer region of primate cone inner segments. This feature has been identified previously, but this was in eyes from AMD patients and the authors regarded this as a feature of degeneration^60^. However, there was no indication that retinae in our older animals were degenerate. Hence, it is likely that this feature is one of ageing. The phenomenon of mitochondrial remodelling and shuffling is to our knowledge not seen in mice. One consequence of the presence of this mitochondrial free region is that it separates a key energy source from the energy consuming outer segment, which may have consequences for photoreceptor function.

The key finding of our study is the impact of age upon ATP production. Improved mitochondrial function can be obtained by exposure to long wavelength light that is absorbed by cytochrome c oxidase, which is the rate limiting enzymatic complex in their respiration. Exposure to these wavelengths improves mitochondrial membrane potential^61^ and ATP production^16,17^ in the retinae of old mice and also improves aged retinal function in mice and insects^62–64^. This improvement may be linked to improved ATP availability to the high energy consuming ionic membrane pumps that are responsible for maintaining a potential difference across the cell membrane. These are known to decline with age^65^. This may also explain the general improvements found in CNS function following exposure to longer wavelengths in both ageing and induced pathology^64,66,67^. As the application of longer wavelengths improves mitochondrial function and ATP production, this may be a productive route for assisting in age related decline in the human retina.

The results of this study may question the validly of some therapeutic approaches targeting aged inflammation and/or amyloid beta deposition in the human retina^68,69^. Many studies use mice to test mode of action of products and their ability to reduce retinal inflammation and clear deposition. They then progress to the use of primates mainly for demonstration of product safety before going into human trials. Hence, the primate is not used as the platform for the original experimentation. While the primates used here are not humans and were maintained on a healthy diet, it is note worth that there has been a lack of general success of clinical trials that have targeted inflammatory agents and deposited material in age related macular degeneration^70–72^ and the reason may partly reside in the use of mice for the basic research used to justify the therapeutic approach.

## Materials and Methods

### Animals

All animal were used with University College London local ethics committee approval and conformed to the United Kingdom Animal Licence (Scientific Procedures) Act 1986. UK Home Office licence number PPL 70/6571. **Primates** Ocular tissues were acquired from a large long established colony of *Macaca fascicularis* maintained Public Health England. The primary purpose of animal usage was different from the aims of this study and eyes were only retrieved after death. Primates were of Mauritian origin and have a smaller gene pool than those from Indonesia and age more rapidly.

Eye retrieval followed sedation with ketamine (4 mg/kg) and an overdose of intravenous sodium pentobarbital (20 mg/kg). The eyes were removed rapidly. One eye from each animal was rapidly dissected removing retina, retinal pigment epithelial (RPE) and choroid from the macular and defined region of the periphery. At each location the retina was separated from RPE and choroid. Samples were taken from the central macular. Those from the periphery were pooled from the nasal, ventral and dorsal regions approximately 3–4 mm distant from the optic nerve head. These samples were rapidly snap frozen and stored at −80 °C for protein arrays. Primate tissues arrays from different retinal regions were derived from individual animals. Tissues from specific regions were pooled across primates. The other eye was fixed in 4% paraformaldehyde for approximately 36–48 h and then transferred to phosphate buffer with 0.025% sodium azide. Samples were taken from 5 young female animals (average age 4 years and 2 months. SD ± 2 months) and 5 old female primates (average age 14 years and 7 months. SD ± 4 months). At 4 years old these animals are at the early stages of sexual maturity. Their eye size is adult like. They display no indication of physical ageing. Their lenses are fully transparent. By 14 years the animals have reduced fertility, are less active and their lenses have reduced transparency. Within this aged cohort there is an increased probability of arthritic development, diabetes and grey body hair. However, all of the aged animals used in this study were healthy and had bi-monthly heath checks by a veterinary office specialising in primate health. Primates in this colony rarely lived beyond 18 years and at 14 years show significant and obvious signs of ageing. Additional eyes from 3 young female and 3 old female primates were dissected and the retinae fixed for scanning EM.

### Mice

C57Bl/6 female mice were used for comparison on protein arrays for cytokines. These were from 6 animals that were 2 months and 6 that were 12 months old. It is open to debate how best to match ageing in mice against that in primate. However, at 2 months the animals are, like the primates, in the early stages of sexual maturity and physically active. By 12 months fertility has declined significantly and there are less active. Over 12 months of age there is a marked increase in cataract development and reduced survival. Mice were killed by cervical dislocation and one eye from each was removed and the retina dissected free and snap frozen as above.

### Protein arrays

In the primate tissue snap frozen retinal samples were either used to assess the levels of cytokine using the Proteome Profiler Human Cytokine Array Panel A (R & D Systems, Minneapolis, MN, USA) or the levels of cell stress-related proteins using the Human Cell Stress Proteome Profiler Array kit (R&D Systems, Minneapolis, MN, USA). While these arrays are for human, at both gene and protein levels there is a 93–99% homology between human and Macaque cytokines^73^. Retinae were pooled from defined retinal regions in different primates and across whole retinae in mice. The retinae were homogenized in RIPA buffer (Millipore, UK) containing protease inhibitors (Sigma, UK). Lysates were centrifuged at 1000 × g for 10 minutes at 4 °C and the supernatant was used for the arrays and were conducted according to the manufacturer’s instructions and offer a parallel determination of 36 cytokines for the cytokine array and 26 for the human cell stress related proteins. Protein concentration was calculated using the Protein Assay (Thermo Scientific). Protein Array Analyzer for Image J was used to quantify and determine spot density. In the mouse tissues snap frozen retinae were used to assess the levels of cytokine using the Proteome Profiler Mouse Cytokine Array Panel A (R & D Systems, Minneapolis, MN, USA).

### ATP measurements

ATP extracts were performed using 6 M guanidine hydrochloride to each frozen neural retina sample, which was then homogenized using a sonicator. The homogenates were briefly frozen on dry ice and then denatured at 95 °C for 5 minutes. Then they were centrifuged at max speed for 10 minutes at 4 °C and the supernatants were transferred into a new centrifuge tube. The samples were diluted 50-fold with deionized water and 10 µL of this diluted extract was used for this analysis. ATP concentration in all extracts was measured by using an ATP determination kit (ThermoFisher scientific, UK).

### Glycolysis measurements

The specific activity of the glyceraldehyde-3-phosphate dehydrogenase was measured modifying the procedures described by Krebs^74^ and Velick^75^ as a metric of glycolysis. The activity of this enzyme was measured by determining the increase in the absorption at 340 nm resulting from the reduction of NAD. Briefly, snap frozen retinal tissue was homogenised in pyrophosphate/arsenate buffer (0.015 M sodium pyrophosphate buffer, pH 8.5 containing 0.03 M sodium arsenate) and total protein content measured with the BCA assay. The reaction mixture contained pyrophosphate/arsenate buffer, supplemented with 0.25 mM NAD, 3 mM DTT. Per sample, 1.5 µg of protein was added to the assay followed by the addition of D-glyceraldehyde-3-phosphate (final concentration of 0.25 mM) to start the reaction. Each group consisted of 5 replicates. Measurements were in duplicate.

### Cytochrome C oxidase activity

The specific activity of complex IV (cytochrome c oxidase) was measured adapting a previously published protocol^76^. Complex IV activity was measured by determining the rate of oxidation of reduced cytochrome c at 550 nm. Briefly, snap frozen retinal tissue was homogenised in isolation buffer (225-mM mannitol, 75-mM sucrose, 10-mM 3-(N-morpholino) propanesulfonic acid, 1-mM ethylene glycol tetra acetic acid, pH 7.2) and total protein measured (BCA assay). The reaction mixture contained 5-mM MgCl2, 2 μg/mL rotenone, 2 μg/mL antimycin A, 1-mM DDM, 45-μM cytochrome c and 0.75 µg protein from each sample. The reaction was inhibited with 4 mM KCN. Sodium dithionite was used to reduce cytochrome c^76^. Each group consisted of 5 replicates. Measurements were in duplicate.

### Immunohistochemistry

Primate eyes were fixed in 4% paraformaldehyde in PBS. After a few days, eyes were washed in PBS, dissected and the anterior tissues removed. A horizontal strip containing the retina, RPE and choroid was removed running from the temporal periphery, through the macular and fovea into the nasal retina. These were cryoprotected in 30% sucrose and then embedded in OCT (Optimal Cutting Temperature compound, VWR UK). Frozen sections were cryosectioned at 10 µm and immunostained with; a mouse monoclonal anti amyloid beta 4G8 (1:100, Covance, USA) and two amyloid antibodies, an anti-beta Amyloid 1–38 antibody EPR1878 (ab108253, Abcam) and beta Amyloid Antibody (DE2B4, Novus Biologicals). Secondary antibodies conjugated with biotin-SP were used accordingly (1:1000, Jackson ImmunoResearch Laboratories, UK) followed by a horseradish peroxidase solution. Chromogenic visualization was achieved with 3,3-diaminobenzidine (DAB, DAKO, USA). Slides were mounted in glycerol and coverslipped. Sections were viewed and images captured using a bright-field microscope (Olympus BX50F4, Olympus, UK) and a Nikon DXM1200 (Nikon, UK) digital camera.

### Resin embedded histology light microscopy

Eyes from 5 young and 5 old primates were dissected and a horizontal strip of the neural retina together with the RPE/choroidal tissues were removed running from the periphery to the macular were processed for resin embedded plastic. They were post-fixed with 2% paraformaldehyde and 2% glutaldehyde in PBS for 24 h, followed by repeated PBS washing and then were fixed in 1% OsO4 in 0.1 M PBS for 2 h. They were then thoroughly washed in distilled water and dehydrated through a graded series of ethanol. Then infiltrated, polymerised and embedded in Technovit 7100 historesin (Taab Laboratories equipment, UK). Resin sections were cut at 5μm, histologically stained with either 1% Toluidine blue or 1% Sudan Black B and mounted in Depex and coverslipped. Measurement of the thickness of the Bruch’s membrane was done using Image J.

### Scanning electron microscopy

Retinae were fixed in 2% paraformaldehyde and 2% glutaldehyde in PBS for 24 h, followed by washing in PBS and then post fixed in 1% osmium tetroxide in 0.1 M PBS for 2 h. Tissues were then thoroughly washed in distilled water and dehydrated through a graded series of ethanol. The specimens were dried with a critical dry point apparatus. After which they were coated with platinum and analysed using a Carl Zeiss scanning electron microscope.

### Measuring the refractive error of photoreceptor outer segments

Small sections of retina previously fixed with 4% paraformaldehyde, were teased apart onto a no.1 glass microscope coverslip in PBS resulting in the release of rod outer segments into the solution. A coverslip was placed over these and sealed with nail vanish.

The optical properties of the photoreceptor outer segments were measured using a digital holographic microscope (DHM) (T1000, LyncéeTec, Lausanne, CH) at a laser wavelength of 660 nm and under a 100x oil immersion objective (Leica, HCX PL Fluotar). The retardation provides a proportional measure of the refractive index of the sample with reference to the surrounding medium. A detailed description of the technique can be found in Colomb *et al.*^77^. Holographic data were reconstructed into 2-dimensional retardation maps from the samples using the DHM software, Koala (version 4) (LyncéeTec, Lausanne, CH) and phase transects calculated across the outer segments. Phase data and statistical analyses were processed using R (version 2.15.3). Values of retardation per micron were recorded from quantitative phase measurements of 10 outer segments from each animal along with diameters of the each cell.

### Statistical analysis

For group comparison a Mann-Whitney U test or a t test was used. Data were analysed using GraphPad Prism version 6.0 for windows (GraphPad, San Diego, USA).

## Supplementary information

Supplementary Dataset 1

## Data Availability

All data are available on request.
