# Supplementary material for: Fundamental differences in patterns of retinal ageing between primates and mice
Source: Sci Rep. 2019 Aug 29;9:12574. doi: 10.1038/s41598-019-49121-0 (PMC6715671; doi:10.1038/s41598-019-49121-0)
Supplement: Supplementary file 1 — Supplementary Dataset 1 [file 41598_2019_49121_MOESM1_ESM.docx]

**Fundamental differences in patterns of retinal ageing between primates and mice.**

**Supplementary Information**

**Jaimie Hoh Kam^1^, Tobias W Weinrich^1^, Harpreet Shinhmar^1^, Michael B Powner^2^, Nicholas W Roberts^3^, Asmaa Aboelnour^4^ and Glen Jeffery^1^**

**^1^University College London, Institute of Ophthalmology EC1V9EL UK, ^2^City, University of London, Centre of Applied Vision Research EC1V0HB, UK, ^3^School of Biological Sciences, University of Bristol BS8 1TQ, UK, ^4^Histology and Cytology Department, Faculty of Veterinary Medicine, Damanhour University, Egypt.**

**Supplementary Data for Figure 1**

|  | **Cell Stress** | | |
| --- | --- | --- | --- |
|  |  | **Primate** | |
|  | **Retina** | **Y** | **O** |
| ADAMTS1 | | 0.0062 | 0.0007 |
| Bcl-2 | | 0.0028 | 0.0014 |
| Carbonic Anhydrase IX | | 0.0007 | 0.0000 |
| Cited-2 | |  | 0.0011 |
| COX-2 | | 0.0026 | 0.0017 |
| Cytochrome C | |  | 0.0032 |
| Dkk-4 | |  | 0.0043 |
| FABP-1 | |  | 0.0043 |
| HIF-1α | | 0.0017 | 0.0041 |
| HIF-2α | | 0.0011 | 0.0019 |
| Prhospho-HPS27 | |  | 0.0006 |
| HSP60 | | 0.0022 | 0.0033 |
| HSP70 | | 0.0332 | 0.0264 |
| ID0 | | 0.0001 | 0.0020 |
| Phospho-JNK Pan | | 0.0015 | 0.0028 |
| NFκB1 | | 0.0029 | 0.0030 |
| p21/CIP1 | | 0.0025 | 0.0047 |
| p27 | | 0.0816 | 0.0574 |
| Phospho-p38a | | 0.0009 | 0.0012 |
| Phoshpo-p53 | | 0.0048 | 0.0002 |
| PON1 | |  | 0.0046 |
| PON2 | | 0.0015 | 0.0020 |
| PON3 | |  | 0.0021 |
| Thioredoxin-1 | | 0.0005 | 0.0013 |
| SIRT2 | | 0.0041 | 0.0067 |

|  | **Cell Stress** | | | | | |
| --- | --- | --- | --- | --- | --- | --- |
|  |  | **Macular** | |  | **Periphery** | |
|  | **Retina** | **Y** | **O** |  | **Y** | **O** |
| ADAMTS1 | | 0.0049 |  |  | 0.0075 | 0.0013 |
| Bcl-2 | |  |  |  | 0.0056 | 0.0027 |
| Carbonic Anhydrase IX | |  |  |  | 0.0015 | 0.0001 |
| Cited-2 | |  | 0.0007 |  |  | 0.0015 |
| COX-2 | | 0.0046 |  |  | 0.0005 | 0.0034 |
| Cytochrome C | |  | 0.0038 |  |  | 0.0026 |
| Dkk-4 | |  | 0.0054 |  |  | 0.0031 |
| FABP-1 | |  | 0.0055 |  |  | 0.0032 |
| HIF-1α | | 0.0033 | 0.0060 |  |  | 0.0021 |
| HIF-2α | | 0.0005 | 0.0011 |  | 0.0017 | 0.0026 |
| Prhospho-HPS27 | |  |  |  |  | 0.0011 |
| HSP60 | | 0.0025 | 0.0046 |  | 0.0019 | 0.0020 |
| HSP70 | | 0.0410 | 0.0417 |  | 0.0254 | 0.0112 |
| ID0 | |  | 0.0013 |  | 0.0003 | 0.0027 |
| Phospho-JNK Pan | | 0.0030 | 0.0029 |  |  | 0.0027 |
| NFκB1 | | 0.0059 | 0.0021 |  |  | 0.0040 |
| p21/CIP1 | | 0.0050 | 0.0021 |  |  | 0.0073 |
| p27 | | 0.0945 | 0.0848 |  | 0.0686 | 0.0299 |
| Phospho-p38a | |  | 0.0011 |  | 0.0019 | 0.0013 |
| Phoshpo-p53 | |  | 0.0000 |  | 0.0096 | 0.0004 |
| PON1 | |  | 0.0020 |  |  | 0.0072 |
| PON2 | |  | 0.0032 |  | 0.0030 | 0.0007 |
| PON3 | |  | 0.0019 |  |  | 0.0023 |
| Thioredoxin-1 | | 0.0010 | 0.0018 |  |  | 0.0007 |
| SIRT2 | | 0.0068 | 0.0062 |  | 0.0015 | 0.0073 |

**Supplementary Figure legend.** The raw data that form the basis of the relative intensity heat maps shown in Figure 1. White regions represent no detectable protein. Other colours follow standard convention.

**Supplementary Data for Figure 2**

**Cytokines in primate retina**

|  | **Primate** | |
| --- | --- | --- |
|  | **Y** | **O** |
| CCL1 | 0.0028 |  |
| CCL2 | 0.0002 |  |
| MIP-1α/β | 0.0006 |  |
| CCL5 |  |  |
| C5/C5a |  |  |
| CXCL1 | 0.0018 | 0.0009 |
| CXCL11 | 0.0009 |  |
| GM-CSF |  |  |
| IL-1α | 0.0015 |  |
| IL-1β | 0.0011 |  |
| IL-1ra | 0.0007 | 0.0004 |
| IL-2 |  |  |
| IL-5 |  |  |
| IL-6 | 0.0000 |  |
| IL-12 p70 | 0.0002 | 0.0004 |
| IL-16 | 0.0005 |  |
| TNF-α | 0.0002 | 0.0003 |
| TREM-1 | 0.0006 |  |
| CXCL10 | 0.0041 | 0.0008 |
| IFN-γ | 0.0011 |  |
| CD54 |  |  |
| CXCL12 | 0.1377 | 0.0884 |
| G-CSF |  |  |
| IL-4 |  |  |
| IL-10 | 0.0016 | 0.0006 |
| IL-13 | 0.0028 | 0.0007 |
| IL-27 | 0.0006 |  |

| CD40 | 0.0007 |  |
| --- | --- | --- |
| IL-8 | 0.0002 |  |
| IL-17A | 0.0029 |  |
| IL-17E | 0.0011 | 0.0004 |
| IL-18 | 0.0006 | 0.0003 |
| IL-32α | 0.0022 | 0.0011 |
| MIF | 0.8755 | 0.8047 |
| IL-21 | 0.0007 | 0.0001 |
| Serpin E1 | 0.0013 | 0.0010 |
|  |  |  |

|  |  | | | | |
| --- | --- | --- | --- | --- | --- |
|  | **Macular** | |  | **Periphery** | |
| **Retina** | **Y** | **O** |  | **Y** | **O** |
| CCL1 | 0.0021 |  |  | 0.0035 |  |
| CCL2 |  |  |  | 0.0004 |  |
| MIP-1α/β |  |  |  | 0.0012 |  |
| CCL5 |  |  |  |  |  |
| C5/C5a |  |  |  |  |  |
| CXCL1 |  |  |  | 0.0036 | 0.0018 |
| CXCL11 |  |  |  | 0.0018 |  |
| GM-CSF |  |  |  |  |  |
| IL-1α |  |  |  | 0.0031 |  |
| IL-1β |  |  |  | 0.0023 |  |
| IL-1ra |  | 0.0004 |  | 0.0013 | 0.0003 |
| IL-2 |  |  |  |  |  |
| IL-5 |  |  |  |  |  |
| IL-6 |  |  |  | 0.0001 |  |
| IL-12 p70 |  |  |  | 0.0003 | 0.0007 |
| IL-16 |  |  |  | 0.0010 |  |
| TNF-α |  |  |  | 0.0004 | 0.0005 |
| TREM-1 |  |  |  | 0.0011 |  |
| CXCL10 |  |  |  | 0.0083 | 0.0016 |
| IFN-γ |  |  |  | 0.0023 |  |
| CD54 |  |  |  |  |  |
| CD40 |  |  |  | 0.0014 |  |
| IL-8 |  |  |  | 0.0004 |  |
| IL-17A |  |  |  | 0.0057 |  |
| IL-17E |  |  |  | 0.0021 | 0.0009 |
| IL-18 |  |  |  | 0.0012 | 0.0006 |
| IL-32a |  |  |  | 0.0043 | 0.0022 |
| MIF | 0.8897 | 0.8204 |  | 0.8612 | 0.7889 |
| CXCL12 | 0.1505 | 0.1189 |  | 0.1249 | 0.0578 |
| G-CSF |  |  |  |  |  |
| IL-4 |  |  |  |  |  |
| IL-10 |  |  |  | 0.0032 | 0.0012 |
| IL-13 | 0.0013 |  |  | 0.0043 | 0.0013 |
| IL-27 |  |  |  | 0.0011 |  |
| IL-21 |  |  |  | 0.0014 | 0.0003 |
| Serpin E1 |  |  |  | 0.0025 | 0.0019 |

**Supplementary Figure legend.** The raw data that form the basis of the relative intensity heat maps shown in Figure 2. White regions represent no detectable protein. Other colours follow standard convention.

**Supplementary Data for Figure 3**

**Cytokines in choroid/RPE**

|  |  | |
| --- | --- | --- |
|  | **Primate** | |
| **Choroid** | **Y** | **O** |
| CCL1 | 0.0070 | 0.0129 |
| CCL2 | 0.0070 | 0.0115 |
| MIP-1α/β | 0.0045 | 0.0097 |
| CCL5 | 0.0112 | 0.0206 |
| C5/C5a | 0.0015 | 0.0044 |
| CXCL1 | 0.0016 | 0.0034 |
| CXCL11 | 0.0074 | 0.0128 |
| GM-CSF | 0.0051 | 0.0106 |
| IL-1α | 0.0020 | 0.0071 |
| IL-1β | 0.0011 | 0.0039 |
| IL-1ra | 0.0211 | 0.0739 |
| IL-2 | 0.0070 | 0.0118 |
| IL-5 | 0.0025 | 0.0029 |
| IL-6 | 0.0011 | 0.0063 |
| IL-12 p70 | 0.0000 | 0.0011 |
| IL-16 | 0.0754 | 0.1137 |
| TNF-α | 0.0045 | 0.0090 |
| TREM-1 | 0.0037 | 0.0083 |
| CXCL10 | 0.0020 | 0.0061 |
| IFN-γ | 0.0002 | 0.0032 |
| CD54 | 0.0019 | 0.0081 |
| CD40 | 0.0044 | 0.0100 |
| IL-8 | 0.0036 | 0.0106 |
| IL-17A | 0.0050 | 0.0064 |
| IL-17E | 0.0028 | 0.0077 |
| IL-18 | 0.0002 | 0.0054 |
| IL-32a | 0.0003 | 0.0028 |
| MIF | 0.6425 | 0.6758 |
| CXCL12 | 0.3293 | 0.3195 |
| G-CSF | 0.0053 | 0.0080 |
| IL-4 | 0.0039 | 0.0064 |
| IL-10 | 0.0000 | 0.0007 |
| IL-13 | 0.0155 | 0.0191 |
| IL-27 | 0.0069 | 0.0042 |
| IL-21 | 0.0025 | 0.0044 |
| Serpin E1 | 0.0334 | 0.0393 |

|  | **Macular** | |  | **Periphery** | |
| --- | --- | --- | --- | --- | --- |
| **Choroid** | **Y** | **O** |  | **Y** | **O** |
| CCL1 | 0.0132 | 0.0132 |  | 0.0008 | 0.0125 |
| CCL2 | 0.0124 | 0.0108 |  | 0.0016 | 0.0122 |
| MIP-1α/β | 0.0080 | 0.0106 |  | 0.0010 | 0.0088 |
| CCL5 | 0.0169 | 0.0209 |  | 0.0056 | 0.0202 |
| C5/C5a | 0.0028 | 0.0043 |  | 0.0002 | 0.0045 |
| CXCL1 | 0.0032 | 0.0043 |  |  | 0.0024 |
| CXCL11 | 0.0120 | 0.0117 |  | 0.0029 | 0.0139 |
| GM-CSF | 0.0069 | 0.0103 |  | 0.0033 | 0.0108 |
| IL-1α | 0.0032 | 0.0093 |  | 0.0008 | 0.0048 |
| IL-1β | 0.0001 | 0.0047 |  | 0.0021 | 0.0032 |
| IL-1ra | 0.0252 | 0.0632 |  | 0.0170 | 0.0846 |
| IL-2 | 0.0120 | 0.0109 |  | 0.0020 | 0.0127 |
| IL-5 | 0.0032 | 0.0053 |  | 0.0017 | 0.0004 |
| IL-6 | 0.0016 | 0.0076 |  | 0.0006 | 0.0051 |
| IL-12 p70 |  | 0.0022 |  | 0.0000 |  |
| IL-16 | 0.0794 | 0.1217 |  | 0.0713 | 0.1058 |
| TNF-α | 0.0074 | 0.0098 |  | 0.0016 | 0.0082 |
| TREM-1 | 0.0055 | 0.0064 |  | 0.0019 | 0.0102 |
| CXCL10 | 0.0040 | 0.0046 |  |  | 0.0076 |
| IFN-γ | 0.0005 | 0.0046 |  |  | 0.0017 |
| CD54 | 0.0027 | 0.0116 |  | 0.0010 | 0.0045 |
| CD40 | 0.0082 | 0.0102 |  | 0.0006 | 0.0098 |
| IL-8 | 0.0043 | 0.0083 |  | 0.0030 | 0.0129 |
| IL-17A | 0.0075 | 0.0090 |  | 0.0026 | 0.0038 |
| IL-17E | 0.0034 | 0.0065 |  | 0.0023 | 0.0089 |
| IL-18 | 0.0003 | 0.0045 |  | 0.0001 | 0.0064 |
| IL-32a |  | 0.0055 |  | 0.0006 |  |
| MIF | 0.5639 | 0.6935 |  | 0.7211 | 0.6580 |
| CXCL12 | 0.2786 | 0.3666 |  | 0.3800 | 0.2724 |
| G-CSF | 0.0086 | 0.0091 |  | 0.0021 | 0.0069 |
| IL-4 | 0.0063 | 0.0057 |  | 0.0015 | 0.0071 |
| IL-10 |  | 0.0013 |  | 0.0000 |  |
| IL-13 | 0.0215 | 0.0204 |  | 0.0096 | 0.0179 |
| IL-27 | 0.0089 | 0.0078 |  | 0.0048 | 0.0005 |
| IL-21 | 0.0004 | 0.0044 |  | 0.0046 | 0.0044 |
| Serpin E1 | 0.0467 | 0.0500 |  | 0.0201 | 0.0285 |

**Supplementary Figure legend.** The raw data that form the basis of the relative intensity heat maps shown in Figure 3. White regions represent no detectable protein. Other colours follow standard convention.

**Supplementary Data for Figure 4**

**Cytokines common to both primate and mouse**

|  |  |  |  |  |  |
| --- | --- | --- | --- | --- | --- |
|  | **Primate** | |  | **Mouse** | |
|  | **Y** | **O** |  | **Y** | **O** |
| CCL1 | 0.0028 |  |  | **0.0560** | **0.0775** |
| CCL2 | 0.0002 |  |  | **0.0755** | **0.0795** |
| MIP-1α/β | 0.0006 |  |  | **0.0595** | **0.0567** |
| CCL5 |  |  |  | **0.0349** | **0.0548** |
| C5/C5a |  |  |  | **0.6817** | **0.6897** |
| CXCL1 | 0.0018 | 0.0009 |  | **0.0437** | **0.0551** |
| CXCL11 | 0.0009 |  |  | **0.2028** | **0.3243** |
| GM-CSF |  |  |  | **0.0713** | **0.8645** |
| IL-1α | 0.0015 |  |  | **0.1602** | **0.2770** |
| IL-1β | 0.0011 |  |  | **0.0236** | **0.0511** |
| IL-1ra | 0.0007 | 0.0004 |  | **0.1296** | **0.2355** |
| IL-2 |  |  |  | **0.0394** | **0.0567** |
| IL-5 |  |  |  | **0.0145** | **0.0178** |
| IL-6 | 0.0000 |  |  | **0.0022** | **0.0168** |
| IL-12 p70 | 0.0002 | 0.0004 |  | **0.0151** | **0.0218** |
| IL-16 | 0.0005 |  |  | **0.0432** | **0.0710** |
| TNF-α | 0.0002 | 0.0003 |  | **0.1268** | **0.1316** |
| TREM-1 | 0.0006 |  |  | **0.0581** | **0.0662** |
| CXCL10 | 0.0041 | 0.0008 |  | **0.0943** | **0.1037** |
| IFN-γ | 0.0011 |  |  | **0.2308** | **0.2673** |
| CD54 |  |  |  | **0.7991** | **0.8967** |
| CXCL12 | 0.1377 | 0.0884 |  | **0.2605** | **0.3149** |
| G-CSF |  |  |  | **0.0852** | **0.1016** |
| IL-4 |  |  |  | **0.0961** | **0.0969** |
| IL-10 | 0.0016 | 0.0006 |  | **0.0170** | **0.0249** |
| IL-13 | 0.0028 | 0.0007 |  | **0.0678** | **0.0937** |
| IL-27 | 0.0006 |  |  | **0.0612** | **0.0767** |

**Cytokines exclusive to either primate or mouse**

|  | **Primate** | |
| --- | --- | --- |
|  | **Y** | **O** |
| CD40 | 0.0007 |  |
| IL-8 | 0.0002 |  |
| IL-17A | 0.0029 |  |
| IL-17E | 0.0011 | 0.0004 |
| IL-18 | 0.0006 | 0.0003 |
| IL-32α | 0.0022 | 0.0011 |
| MIF | 0.8755 | 0.8047 |
| IL-21 | 0.0007 | 0.0001 |
| Serpin E1 | 0.0013 | 0.0010 |

|  | **Mouse** | |
| --- | --- | --- |
|  | **Y** | **O** |
| CCL11 | 0.0236 | 0.3272 |
| IL-7 | 0.1148 | 0.1413 |
| M-CSF | 0.0895 | 0.1028 |
| IL-17 | 0.0710 | 0.1023 |
| CXCL9 | 0.0666 | 0.0893 |
| IL-23 | 0.0539 | 0.0810 |
| CCL17 | 0.0498 | 0.0521 |
| CXCL2 | 0.0343 | 0.0415 |
| CCL12 | 0.0412 | 0.0395 |
| CCL4 | 0.0353 | 0.0394 |
| IL-3 | 0.1777 | 0.2285 |
| CXCL13 | 0.2622 | 0.2182 |
| TIMP-1 | 0.4055 | 0.4767 |

**Supplementary Figure legend.** The raw data that form the basis of the relative intensity heat maps shown in Figure 4. White regions represent no detectable protein. Other colours follow standard convention.
